# Impact of *Eimeria meleagrimitis* and intermittent amprolium treatment on performance and the gut microbiome composition of Turkey poults

**DOI:** 10.3389/fvets.2023.1165317

**Published:** 2023-06-01

**Authors:** Carolina Trujillo-Peralta, Juan David Latorre, Jianmin Chai, Roberto Senas-Cuesta, Aaron Forga, Makenly Coles, Jiangchao Zhao, Xochitl Hernandez-Velasco, Guillermo Tellez-Isaias, John Barta, Lisa Bielke, Billy Hargis, Danielle Graham

**Affiliations:** ^1^Department of Poultry Science, University of Arkansas Division of Agriculture, Fayetteville, AR, United States; ^2^School of Life Science and Engineering, Foshan University, Foshan, China; ^3^Department of Animal Science, Division of Agriculture, University of Arkansas, Fayetteville, AR, United States; ^4^Departamento de Medicina y Zootecnia de Aves, Facultad de Medicina Veterinaria y Zootecnia, UNAM, Mexico City, Mexico; ^5^Department of Pathobiology, Ontario Veterinary College, University of Guelph, Guelph, ON, Canada; ^6^Department of Animal Sciences, The Ohio State University, Wooster, OH, United States

**Keywords:** *Eimeria*, coccidiosis, vaccination, turkey, microbiome, amprolium

## Abstract

**Introduction:**

Drug-sensitive live coccidiosis vaccines have been used to control coccidiosis and renew drug sensitivity in commercial chicken operations. However, only limited species coverage vaccines have been available for commercial turkey producers. This study aimed to assess the effect of an *E. meleagrimitis* vaccine candidate, with and without amprolium intervention, on performance and oocyst shedding. Additionally, the effect of vaccination, amprolium treatment, and *E. meleagrimitis* challenge on intestinal integrity and microbiome composition was evaluated.

**Methods:**

Experimental groups included: (1) NC (non-vaccinated, non-challenged control); (2) PC (non-vaccinated, challenged control); (3) VX + Amprol (*E. meleagrimitis* candidate vaccine + amprolium); and 4) VX (*E. meleagrimitis* candidate vaccine). For VX groups, 50% of the direct poults were orally vaccinated at DOH with 50 sporulated *E. meleagrimitis* oocysts and were comingled with contact or non-vaccinated poults for the duration of the study. From d10-14, VX + Amprol group received amprolium (0.024%) in the drinking water. All groups except NC were orally challenged with 95K *E. meleagrimitis* sporulated oocysts/mL/poult at d23. At d29, ileal and cecal contents were collected for 16S rRNA gene-based microbiome analysis.

**Results and Discussion:**

VX did not affect performance during the pre-challenge period. At d23-29 (post-challenge), VX groups had significantly (*P* < 0.05) higher BWG than the PC group. Contacts and directs of VX groups in LS had significantly reduced compared to PC. As anticipated, amprolium treatment markedly reduced fecal and litter OPG for the VX + Amprol group compared to the VX group which did not receive amprolium. The ileal and cecal content results showed that the PC group had different bacterial diversity and structure, including alpha and beta diversity, compared to NC. Linear discriminant analysis Effect Size (LEfSe) identified that *Lactobacillus salivarius* (ASV2) was enriched in PC’s ileal and cecal content. Compared to NC and PC, the vaccinated groups showed no distinct clusters, but there were similarities in the ileal and cecal communities based on Bray-Curtis and Jaccard distances. In conclusion, these results indicate that vaccination with this strain of *E. meleagrimitis*, with or without amprolium intervention, caused a very mild infection that induced protective immunity and challenge markedly affected both the ileal and cecal microbiome.

## 1. Introduction

*Eimeria,* a genus of obligate intracellular protozoa, cause intestinal coccidiosis in many vertebrate hosts including poultry. Host intestinal epithelial cells are invaded and destroyed by these enteric parasites, impacting gut homeostasis and performance (1). For nearly a century, chemoprophylaxis has been employed to control coccidiosis in commercial poultry (2). Nevertheless, *Eimeria* spp. have been shown to develop resistance to anticoccidial drugs (3). Anticoccidial rotation and shuttle programs have extended the period of use for some drugs by delaying anticoccidial resistance (4). Live vaccination with drug-susceptible *Eimeria* spp. possibly displaces drug-resistant wild-type *Eimeria* strains in the barn environment (4). Application of live coccidiosis vaccine followed by delayed anticoccidial intervention in the feed or drinking water, a program called bioshuttle, permits the development of immunity and improves performance compared to ionophore treatment alone (5). Thus, an efficacious coccidiosis control program would incorporate judicious use of anticoccidial drugs and vaccination to prevent selection of drug-resistant phenotypes in the field.

There are seven *Eimeria* species that infect domestic turkeys (*Meleagris gallopavo* [var. domesticus]) that have been characterized (6). Of those, *E. meleagrimitis, E. adenoeides, E. gallopavonis,* and *E. dispersa* have been shown to cause clinical disease in commercial turkeys (7). Infection with multiple *Eimeria* spp. makes it challenging to predict the impact a single species has at the flock level. A live coccidiosis vaccine containing only two species, *E. meleagrimitis* and *E. adenoeides*, is the only vaccine approved for use in turkeys. There is no evident cross-protection between *Eimeria* species infecting turkeys (8). As a result, an optimal vaccine formulation to displace the drug-resistant wild-type *Eimeria* spp. would consist of those currently affecting the farm. Vaccination with drug-sensitive strains would be the most cost-effective option available to shift the population of *Eimeria* oocysts from pan-resistant to pan-sensitive in a flock. Tailoring a vaccine to contain only the species relevant to a particular complex would be ideal to avoid introducing non-relevant strains. Since *Eimeria* spp. are also prevalent in wild turkey populations (9) and the probability of exposure to anticoccidials is low, *Eimeria* spp. recovered from wild turkeys should be evaluated as potential vaccine candidates. Live vaccination can negatively affect performance and amprolium has been briefly applied to the drinking water to reduce vaccine-related effects without disrupting immune development (7). However, it appears that the timing of application post-vaccination should be selected based on oocyst cycling to not impede immunity development (10).

At present, there are no reports on the impact of live coccidiosis vaccination and/or intermittent amprolium intervention on the intestinal microbiome or gut integrity in turkeys. The complex interactions between the host and the gut microbiome affect digestion and the overall health of the host (11). Consequently, the replication of *Eimeria* spp. within the host may shift the microbiome’s composition and increase intestinal permeability. These effects may be directly or indirectly related. A serum biomarker, fluorescein isothiocyanate-dextran, also known as FITC-d, has been used to assess gastrointestinal permeability in necrotic enteritis and coccidiosis models in chickens (12, 13). The relationship between live coccidiosis vaccination with and without amprolium intervention and its effect on intestinal integrity and the gut microbiome post-challenge with *E. meleagrimitis* has not been evaluated.

In a previous study, our group collected wild turkey fecal samples and generated single oocyst-derived stocks for five of the major *Eimeria* spp. relevant to commercial turkeys: *E. meleagrimitis, E. dispersa, E. meleagridis, E. gallopavonis,* and *E. adenoeides*. *Eimeria meleagrimitis,* one of the more pathogenic species that infect turkeys (14). *Eimeria meleagrimitis* was also the most prevalent species detected in commercial turkey flocks in the midwestern United States (15). Although not all turkey *Eimeria* spp. induce clinical disease, flock performance can be severely impacted and must be considered when vaccinating commercial turkey flocks, especially if additional *Eimeria* spp. have been detected in previous flocks. This current investigation aimed to assess the protective efficacy of a wild turkey-derived, anticoccidial-sensitive (monensin, zoalene, and amprolium) *E. meleagrimitis* vaccine candidate obtained from wild turkeys against homologous challenge. Furthermore, the effect of amprolium intervention, vaccination, and/or challenge on the gut microbiome and intestinal permeability was assessed.

## 2. Materials and methods

### 2.1. Eimeria meleagrimitis

Previously, a drug sensitive strain of *E. meleagrimitis* was recovered from wild turkey feces collected in Maine, United States in 2019. Methods used to isolate, speciate, and characterize the *E. meleagrimitis* used in the current study have been described (16). A single oocyst-derived stock was generated, identity confirmed (PCR and sequencing), and used for vaccination and challenge in the present study. The stock was stored at 4°C in a 2.5% potassium dichromate (PDC; Sigma-Aldrich Co.) solution for >3 months before use.

### 2.2. Preparation of vaccine and challenge stocks

Stocks used for vaccination or challenge were prepared 24 h prior to use. Freshly sporulated *E. meleagrimitis* oocysts were centrifuged at 1300 × g for 10 m to remove the PDC solution. The supernatant was discarded, and the pelleted oocysts were resuspended in 0.9% sterile saline. A McMaster chamber was used to determine the concentration of the stock solution or sporulated oocysts/mL (17). For vaccination, oocysts were prepared to achieve a final concentration of ~50 sporulated *E. meleagrimitis* oocysts/0.25 mL/poult and a challenge concentration of ~95,000 sporulated *E. meleagrimitis* oocysts/1 mL/turkey.

### 2.3. Amprolium

Amprolium (Amprol® 9.6% Oral Solution: Huvepharma), a synthetic anticoccidial, was administered in the drinking water at a concentration of 0.024% from d10-d14 per manufacturer’s guidelines. Only the VX + Amprol received amprolium treatment.

### 2.4. Experimental design

**Figure 1:**
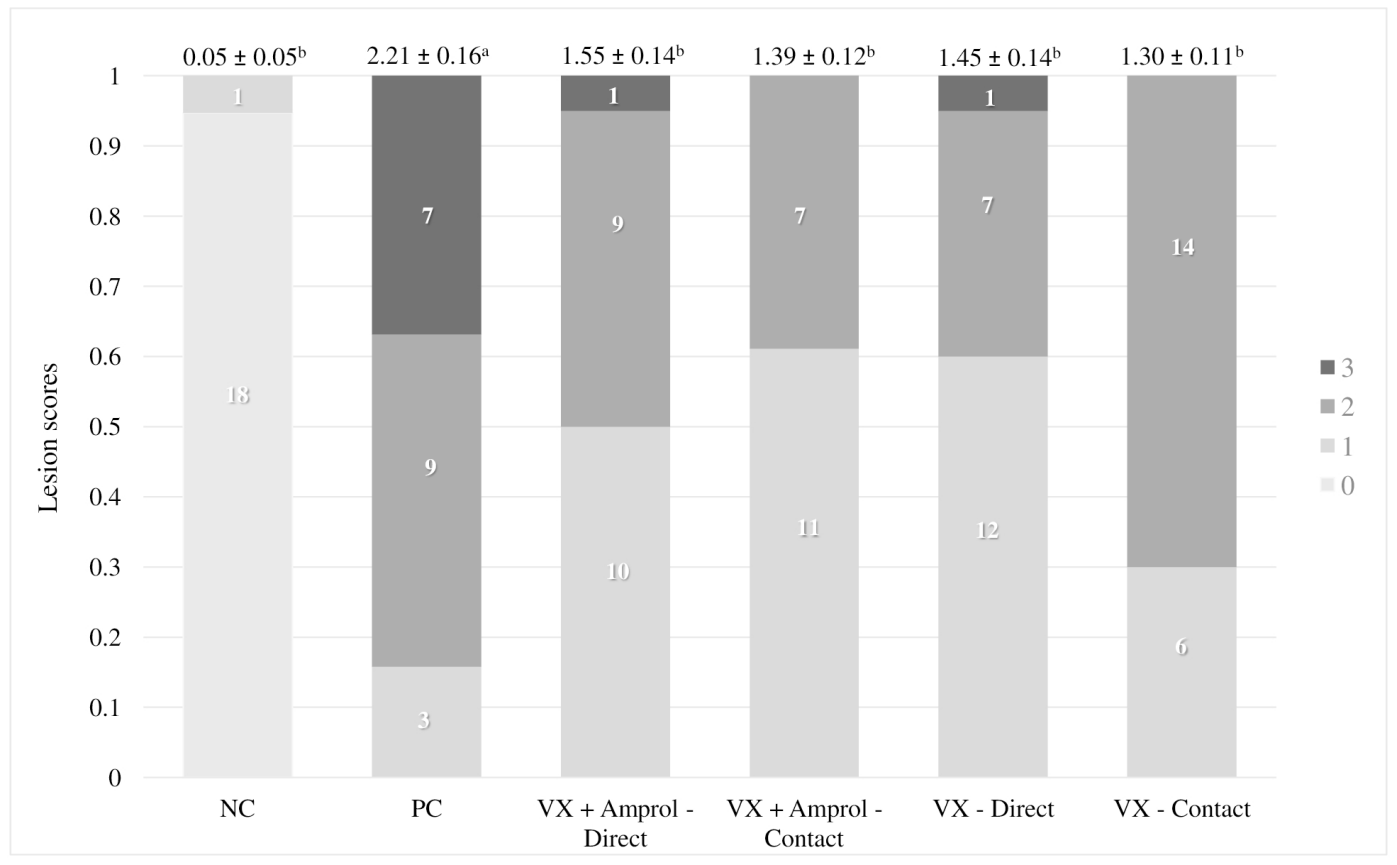
Graphical representation of the experimental timeline and the fluorescein isothiocyanate dextran (FITC-d) administration and general overview of FITC-d assay methodology (Created with BioRender.com).

The experimental design is presented at the top in Figure 1. A total of 280 day-of-hatch (DOH) female turkey poults (Nicholas genetics) were obtained from a commercial hatchery. Poults were individually neck-tagged and randomly allocated into the following treatment groups: (1) NC (non-vaccinated, non-challenged control), (2) PC (non-vaccinated, challenged control), (3) VX + Amprol (*E. meleagrimitis* candidate vaccine + amprolium), and (4) VX (*E. meleagrimitis* candidate vaccine). For the vaccinated groups, 50% of the poults (referred to as directs) received 50 sporulated *E. meleagrimitis* (VX) oocysts/0.25 mL/poult *via* oral gavage immediately prior to placement. At placement, the directs were commingled with non-vaccinated (contacts) poults for the duration of the study. The NC, PC, and contacts of the vaccinated group did not receive any treatment before placement. At d23, turkeys in all groups, excluding the NC group, were challenged with ~95,000 sporulated *E. meleagrimitis* oocysts/1 mL/turkey by oral gavage. Individual body weights (BW) were recorded at DOH, d8, d23, and d29 (termination) to determine average body weight gain (BWG).

Each treatment group was housed in a single 7x7ft floor pen with fresh pine shavings (*n* = 70 poults/pen). Poults were housed in a section of the pen from DOH-d10 to simulate commercial brooding density (0.475 sq. ft./poult). A standard cardboard barrier was used to segregate the poults from the entirety of the pen during the first 10 days. At d10, the barrier was removed, and poults were reared at a density of 0.817 sq. ft./poult from d10-d29 (termination). Litter moisture was maintained in each pen using a generic garden pump sprayer every morning from d2-d7. No additional modifications were made to the litter for the duration of the study. All turkey poults were provided feed and water *ad libitum* throughout all experiments. The lighting program followed management guidelines for commercial turkey hens (18). All animal handling procedures complied with the Institutional Animal Care and Use Committee (IACUC protocol #21117) of the University of Arkansas.

### 2.5. Fecal and litter OPG

Fecal and litter samples were collected from d5-d28 post-vaccination to assess oocyst shedding. Individual fecal samples were collected from a subset of the directs and contacts in the vaccinated groups (*n* = 10 individual samples from d5-d6; *n* = 1 pooled sample at d7; *n* = 5 individual samples at d8; and *n* = 3 individual samples from d9-d28). The difference in the number of individual fecal samples collected throughout the duration of the experiment was due to the sheer amount of time it took to collect the samples. Initially, the goal was to collect *n* = 10 individual samples per group and vaccination level. However, *n* = 3 individual samples were a more feasible number to collect. Pooled fecal samples were collected for the NC and PC group (*n* = 3) from d5–d29. For each treatment group, pooled litter samples were collected from random locations in the pen (*n* = 3). To determine fecal and litter oocysts per gram of feces (OPG), all fecal and litter samples were collected in 5 mL microcentrifuge tubes or 50 mL polypropylene centrifuge tubes, respectively, and then weighed and suspended in 2.5% PDC at a final concentration of 1:2 (w/v). Fecal or litter samples were processed to determine OPG counts using a McMaster counting chamber using a standard formula that includes the initial weight of the sample, volume of saturated NaCl solution, and any additional dilutions that were required (17, 19, 20).

### 2.6. Lesion scores

At d29, or 6d post-homologous challenge, the duodenum to the lower intestine was evaluated post-mortem for each group and vaccination level (*n* = 18–20) to evaluate macroscopic lesions using methods similar to El-Sherry et al. (21) and Gadde et al. (22). Lesions were scored from 0 to 4: “0” represents a healthy organ, whereas a score of “4” represents severe coccidiosis.

### 2.7. Serum fluorescein isothiocyanate dextran

FITC-d (ng/mL) was used as a biomarker to evaluate intestinal permeability as described by Baxter et al. (23) and the methodology has been presented at the bottom in Figure 1. At the end of the trial, turkey poults (*n* = 18–20 for NC and PC; *n* = 13–15 for vaccinated groups) were orally gavaged with 8.32 mg of FITC-d per kg of body weight (FITC-d, MW 3–5 KDa; Sigma-Aldrich Co). One hour after FITC-d administration, turkeys were euthanized by CO_2_ inhalation. Blood samples were collected from the femoral vein and centrifuged (1,000 × g for 10 m at 4°C) to separate the serum.

### 2.8. Microbiome

Ileal and cecal contents were collected from 29-day-old turkey poult hens (*n* = 6/treatment). Samples were stored at-20°C in an RNA/DNA (Zymo Research, Irvine, CA, United States) shield until DNA extraction was performed. Total genomic DNA of ileal and cecal content samples was extracted using the DNeasy PowerLyzer PowerSoil Kit (Qiagen, Germantown, MD, United States) according to the manufacturer’s protocol. The concentration of DNA was measured using a NanoDrop One spectrophotometer (Thermo Fisher Scientific, Madison, WI, United States) and diluted to 10 ng/μL with DNase/RNase-free water. The V4 region of the 16S rRNA gene was amplified using primer sequences (forward: 5′-GTGCC AGCMGCCGCGGTAA-3′ and reverse: 5′-GGACTACHVGGG TWTCTA AT-3′) attached with gene-specific Illumina adapters for each sample (24). PCR amplification was performed using a T100 thermal cycler (Bio-Rad, Hercules, CA, United States). All 16S PCRs conditions consisted of a 30s initial denaturation at 95°C: 30 cycles at 95°C for 10s, annealing at 55°C for 30s, extension at 72°C for 60s, and a final extension at 72°C for 10 m. The PCR products were determined on a 1% agarose gel and normalized using a SequalPrepTM Normalization Plate Kit (Invitrogen, Carlsbad, CA, United States) according to the manufacturer’s recommendations. All purified PCR amplicons were pooled to generate a sequencing library (25). After concentration, the quality of the library was confirmed by KAPA Illumina Library Quantification Kits (Roche, Indianapolis, IN, United States) *via* a quantitative PCR (qPCR, Eppendorf, Westbury, NY, United States) assay and an Agilent 2,100 Bioanalyzer System (Agilent, Santa Clara, CA, United States).

The library was sequenced on a MiSeq sequencer (MiSeq Reagent Kit v2, 500 cycles; Illumina, San Diego, CA, United States). To detect any contamination, a mock community (ZymoBIOMICS™ Microbial Community Standard; Zymo, Irvine, CA, United States), a negative control for DNA, and a negative control for PCR amplification were included in sequencing. Sequencing files obtained from the Illumina sequencer were pre-processed, quality filtered (*Q* > 30), and analyzed using the QIIME2 (2021.4 release) software (26). Deblur algorithm was used for sequence trimming, denoising, chimera removal, and features binning at the amplicon sequence variants (ASV) level (27). Naïve Bayes classifier was employed for the assignment of all sequences into bacterial taxonomy using the Greengenes (v13_8 clustered at 99% identity) reference database. The raw data are available in the NCBI SRA database with the BioProject ID PRJNA.

### 2.9. Statistical analysis

All data were subjected to analysis of variance (ANOVA) as a completely randomized design using JMP Pro 14 software. Significant differences among the means were determined by Tukey’s multiple comparison test for BW, BWG, and serum FITC-d, where statistically significant differences between the means were set at *p* < 0.05. The LS data were determined using Proc Mixed Analysis by SAS 9.4 at *p* < 0.0001. Oocysts per gram of feces (OPG) and litter OPG data were expressed as mean using JMP Pro 14 software.

Alpha diversity, including the Shannon Index and the number of Observed ASVs, was compared using a two-tailed Wilcoxon signed-rank test between two groups (*p* < 0.10). Beta diversity based on Bray-Curtis and Jaccard distances was tested using an analysis of similarity (ANOSIM). The outputs of diversity were visualized using the “ggplot2” package in *R* (v4.1.2). The linear discriminant analysis (LDA) effect size (LefSe), an analytical tool for discovering and interpreting biomarkers of high-dimensional data, was used to identify the signature bacteria associated with the growth stages and intestinal segments. LDA score>2 was used as a criterion for judging the significant effect size (28). The signature bacteria were visualized in a heat map using the “pheatmap” function in R.

## 3. Results

### 3.1. Performance

For average BW, there were no significant differences between all groups at DOH or d8 (Table 1). However, there were significant (*p* < 0.05) differences in BW at d23 only between PC and VX + Amprol – contact, with the VX group having the lower BW at d23. The BW at d29 showed that VX – contact had a markedly higher value than PC with significant (*p* < 0.05) differences; in contrast, the other groups evaluated had no significant differences.

**Table 1:** Effect of *Eimeria meleagrimitis* vaccination, with and without amprolium intervention, and/or *E. meleagrimitis* challenge on average body weight (BW), body weight gain (BWG), lesion scores (LS), and serum FITC-d in turkey poults.

Treatment	NC	PC	VX + Amprol direct	VX + Amprol contact	VX direct	VX contact
BW (g)^1^						
DOH	57.96 ± 0.61	58.31 ± 0.57	57.63 ± 0.77	59.23 ± 0.79	57.00 ± 0.62	57.29 ± 0.68
d8	154.86 ± 2.41	158.28 ± 2.12	163.11 ± 3.66	157.50 ± 3.37	159.00 ± 2.54	155.31 ± 4.84
d23	535.08 ± 7.71^a^^b^	559.81 ± 8.83^a^	524.70 ± 11.62^a^^b^	515.33 ± 12.23^b^	533.43 ± 11.58^a^^b^	536.40 ± 9.70^a^^b^
d29	730.60 ± 11.92^a^^b^	706.60 ± 11.90^b^	750.83 ± 20.55^a^^b^	736.47 ± 18.44^a^^b^	764.73 ± 16.63^a^^b^	783.73 ± 13.67^a^
BWG (g)^1^						
DOH-d8	96.84 ± 2.27	99.97 ± 2.02	105.49 ± 3.47	98.68 ± 3.19	102.00 ± 2.38	98.03 ± 2.46
DOH-d23	477.00 ± 7.56^a^^b^	501.17 ± 8.75^a^	467.07 ± 11.44^a^^b^	456.47 ± 12.05^b^	476.33 ± 11.58^a^^b^	478.80 ± 9.53^a^^b^
DOH-d29	672.52 ± 11.74^a^^b^	648.00 ± 11.78^b^	693.20 ± 16.11^a^^b^	677.60 ± 14.79^a^^b^	707.63 ± 12.88^a^^b^	726.13 ± 10.20^a^
d23–d29	195.52 ± 5.40^b^	145.05 ± 5.10^c^	226.13 ± 9.85^a^	221.13 ± 7.01^a^	231.30 ± 566^a^	247.33 ± 5.24^a^
LS^2^	0.05 ± 0.05^c^	2.21 ± 0.16^a^	1.39 ± 0.12^b^	1.55 ± 0.14^b^	1.30 ± 0.11^b^	1.45 ± 0.14^b^
FITC-d [ng/mL]^1^	141.37 ± 29.78^a^^b^^c^	269.74 ± 25.25^a^	206.61 ± 11.69^a^^b^^c^	250.11 ± 32.87^a^^b^	74.72 ± 29.89^b^^c^	65.38 ± 58.77^c^

Data are expressed as the mean ± standard error.

a Different superscripts indicate significant differences between the treatments at *p* ≤ 0.05.

b Different superscripts indicate significant differences between the treatments at *p* ≤ 0.05.

c Different superscripts indicate significant differences between the treatments at *p* ≤ 0.05.

1 Statistical evaluation using ANOVA followed by *post hoc* Tukey’s range test.

2 Statistical differences between lesion scores detected using SAS proc mixed analysis.

BW: body weight; BWG: body weight gain; LS: lesion scores; FITC-d: fluorescein isothiocyanate dextran.

There were no significant differences in average BWG from DOH-d8 across all groups. The DOH-d23 BWG (pre-challenge BWG) was significantly (*p* < 0.05) higher in PC compared to contacts in VX + Amprol group. However, the post-challenge (d23-d29) BWG of the PC group was significantly (*p* < 0.05) reduced compared to NC and vaccinated groups. Although d23-d29 BWG was considerably (*p* < 0.05) increased for vaccinated groups compared to PC and NC, DOH-d29 BWG was only significantly (*p* < 0.05) different between the PC and VX – contact groups.

### 3.2. Lesion scores and serum FITC-d

**Figure 2:**
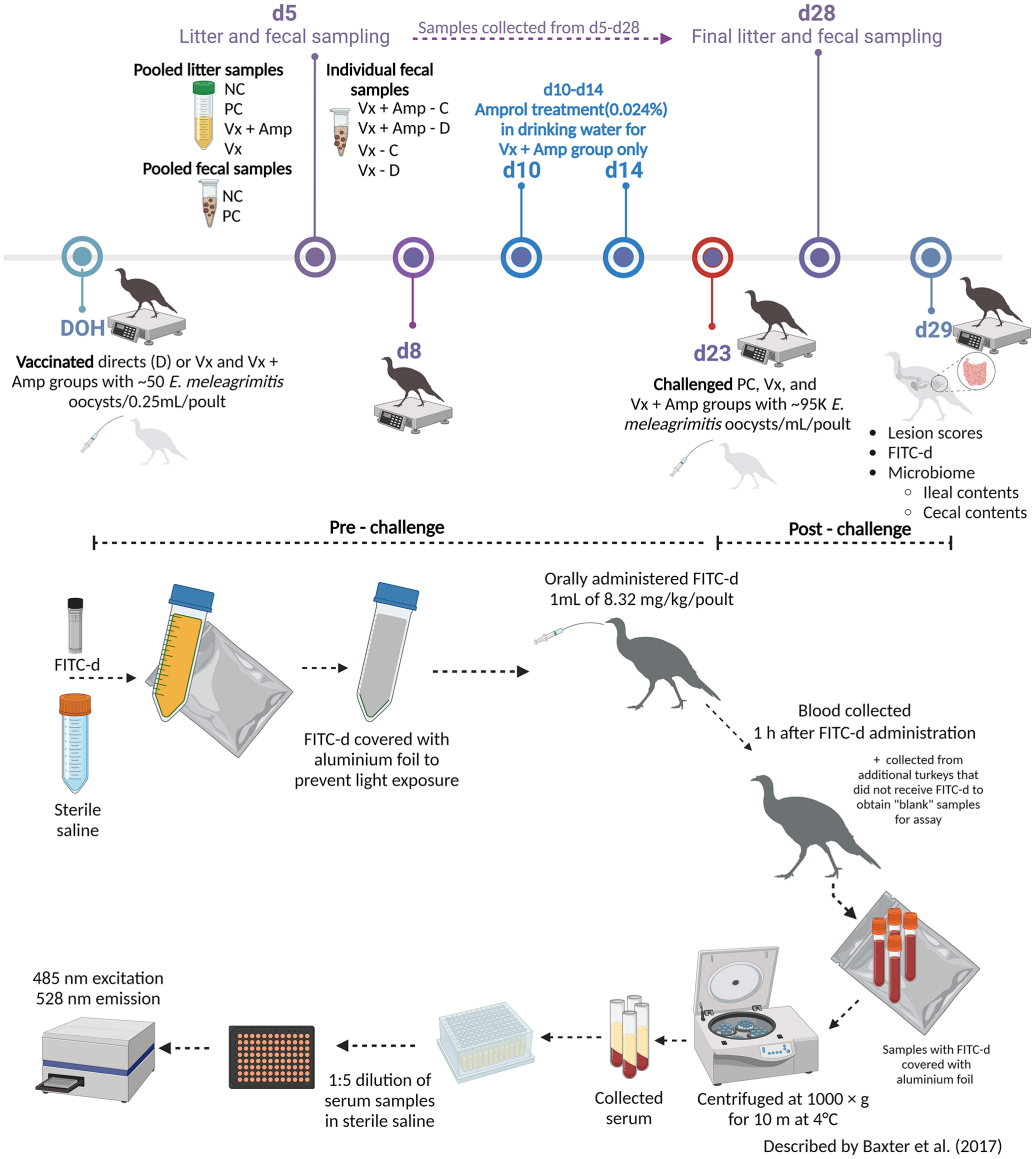
Cumulative lesion scores 6 days post-challenge. At day 23, all poults, except for the NC, were orally challenged with *E. meleagrimitis* (95,000 sporulated oocysts/mL). Six days post-challenge (day 29), a subset of the poults from each group and vaccination level (*n* = 18-20/group) were lesion scored. A lesion score of “0” represents a healthy intestinal tract whereas a score of “4” represents severe coccidiosis. No lesion scores of 4 were observed. Numbers within columns indicate the number of poults evaluated for each lesion score (0–3). Mean lesion score ± standard error presented above columns. Means further separated using Proc Mixed Analysis (SAS 9.4). ^a–c^Different superscripts between treatment groups indicate means differ significantly (*p <* 0.05) (Created with Biorender.com).

At d23, all turkeys, except for the NC, were orally challenged with *E. meleagrimitis* (95,000 sporulated oocysts/mL). After 6d post-challenge, intestinal lesion scores were evaluated using methods similar to El-Sherry et al. (21) and Gadde et al. (22). No scores of 4 were observed in the current study. Lesion scores were significantly (*p* < 0.0001) reduced in direct and contact of VX and VX + Amprol than in PC (Table 1; Figure 2). The average of lesion scores in the vaccinated level group did not have significant differences. The distribution of lesion scores at d29 has been presented in Figure 2. The vaccinated and NC groups had less severe lesion scores than the PC group. Serum FITC-d levels in the PC group were significantly (*p* ≤ 0.05) higher than VX – direct and contact (Table 1). Additionally, serum FITC-d levels for the VX + Amprol – contact group were significantly (*p* < 0.05) higher compared to the VX – contact group.

### 3.3. Fecal and litter OPG

**Figure 3:**
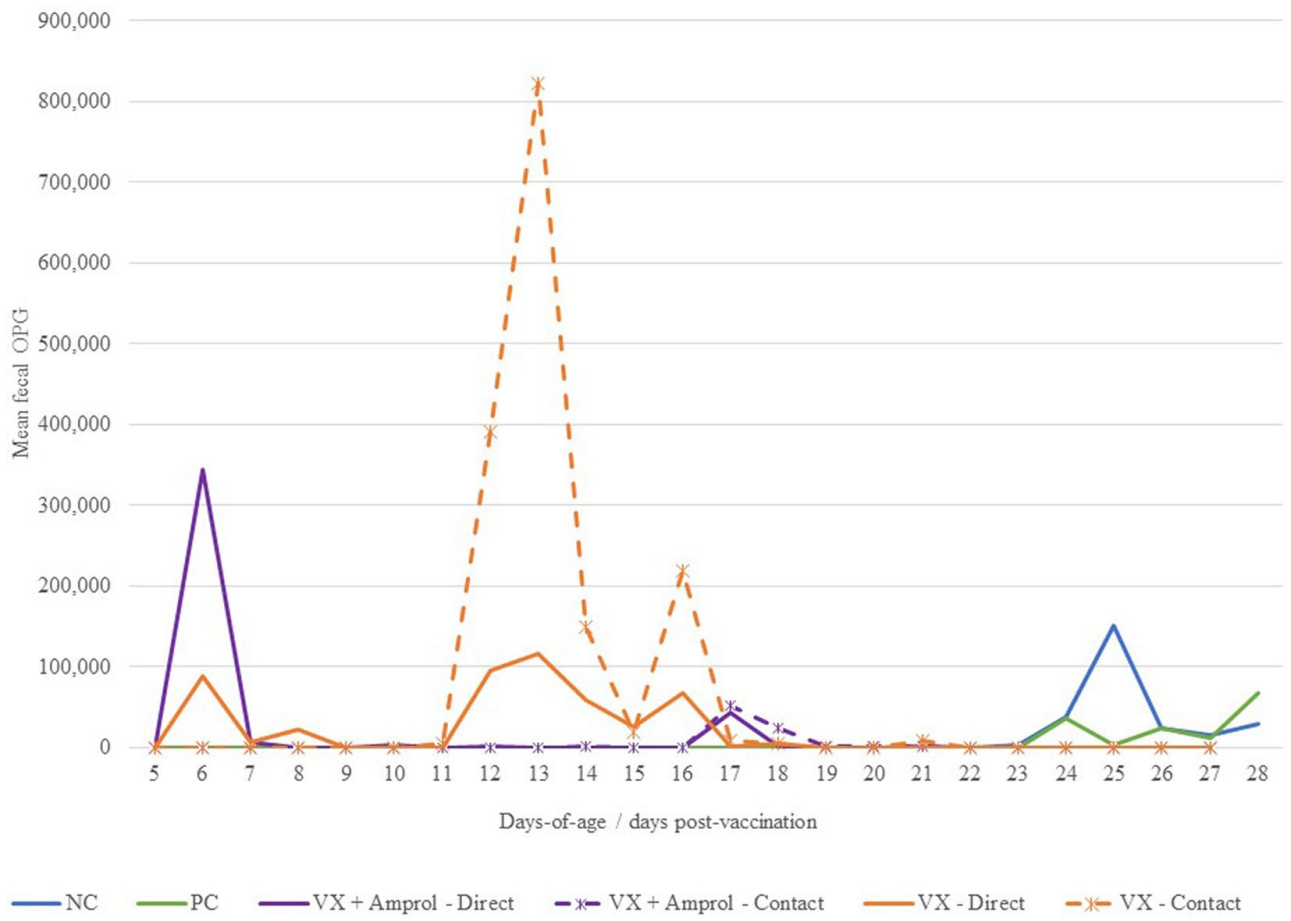
Effect of *E. meleagrimitis* vaccination and/or challenge with and without amprolium intervention on mean fecal oocyst per gram (OPG). For fecal OPG, individual fecal samples were collected from the direct and contact poults (*n* = 3–10 individual fecal samples/group/vaccination level/day) and pooled fecal samples were collected for NC and PC. At DOH, 50% of the poults in the vaccinated groups orally received 50 sporulated *E. meleagrimitis* (VX) oocysts immediately prior to placement. The NC, PC, and contacts did not receive any treatment before placement. VX + Amprol group received amprolium in the drinking water from d10-d14 at 0.024%. At d23, turkeys were orally challenged with *E. meleagrimitis* (95,000 oocysts/mL) except for negative control (NC).

There was a sharp increase in fecal OPG at d6 for turkeys that received 50 *E. meleagrimitis* oocysts at DOH compared to those that did not directly receive the vaccination (Figure 3). From d10-14, the VX + Amprol group received amprolium in the drinking water which reduced fecal OPG for both the direct and contact poults for this period (Figure 3). Alternatively, the naïve contact poults that did not receive any drug intervention to attenuate oocyst cycling had a sharp increase in fecal OPG from d11-d15 compared to all other groups. The importance of proper coccidiosis vaccination methods was represented by the difference in fecal OPG when comparing the trend in the contact of VX group demonstrated that delayed exposure to the vaccine leads to much higher fecal OPG compared to those directly vaccinated at hatch, and those that were treated with amprolium of the directs and contacts. The greatest peak in fecal OPG for the PC was at d24, which was ~24 h post-challenge with *E. meleagrimitis* (95,000 sporulated oocysts/mL). Oocysts were detected in the feces and litter of the NC group at d24 and d25. This was unexpected and suggested that there was low-level cross-contamination likely prior to the challenge period.

**Figure 4:**
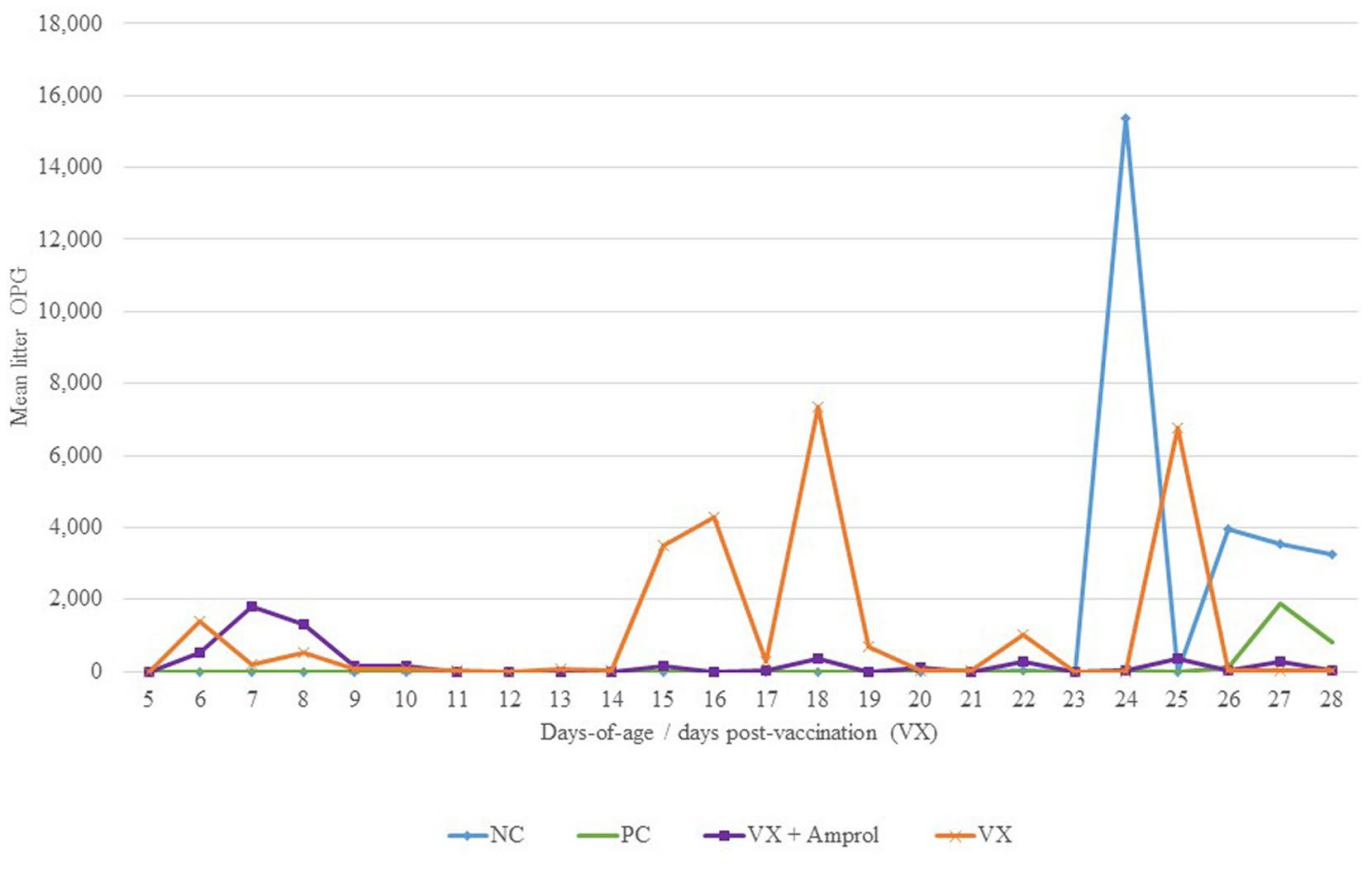
Effect of *E. meleagrimitis* vaccination and/or challenge with and without amprolium intervention on mean litter OPG. Pooled litter samples were collected for each treatment group (*n* = 3). At DOH, 50% of the poults in the vaccinated groups orally received 50 sporulated *E. meleagrimitis* (VX) oocysts immediately prior to placement. The NC, PC, and contacts did not receive any treatment before placement. VX + Amprol group received amprolium in the drinking water from d10-d14 at 0.024%. At d23, turkeys were orally challenged with *E. meleagrimitis* (95,000 oocysts/mL) except for negative control (NC).

For litter OPG (Figure 4), there were differences in peaks between vaccinated groups associated with the administration of amprolium from d10-d14. For instance, the group that did not get drug intervention to attenuate oocyst cycling (VX group) had multiple spikes in litter OPG after d14, whereas the VX + Amprol group had more uniform litter OPGs with only a single sharp increase between d6-d9 (Figure 4).

### 3.4. Microbiome

**Figure 5:**
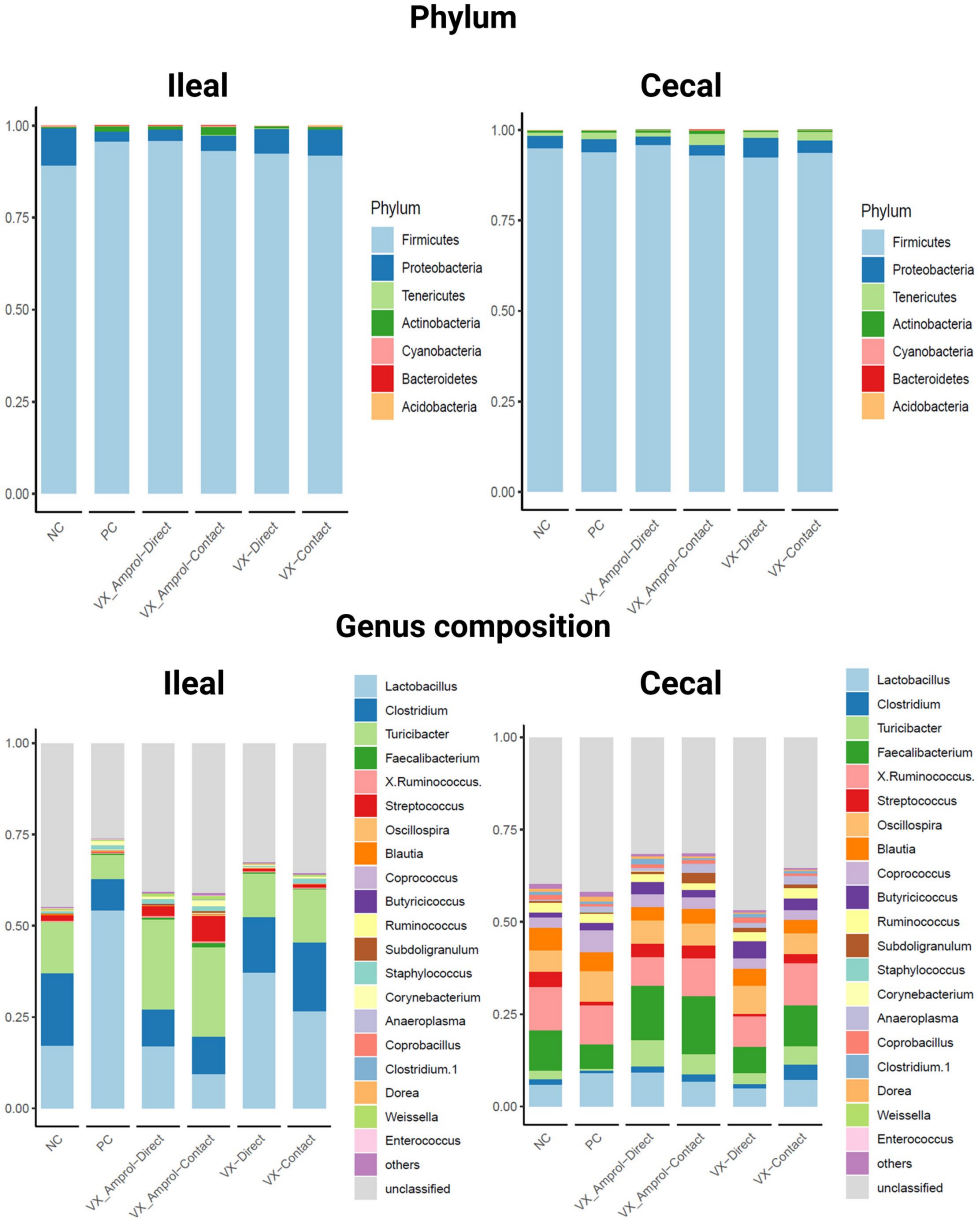
Effect post-challenge of *Eimeria meleagrimitis* vaccination with and without amprolium intervention on phylum and genus composition in the ileum and cecal contents. All groups, except the NC group, were challenged with 95,000 sporulated *E. meleagrimitis* sporulated oocysts at d23. At d29, or 6d post-challenge, ileal and cecal contents were collected from 29-day-old turkey poult hens, *n* = 6/treatment/vaccination level (Created with BioRender.com).

Figure 5 shows the phylum and genus composition in the ileal and cecal content by group. At the phylum level, Firmicutes (89.3–95.9%) was the most dominant taxa, followed by Proteobacteria (2.7–10.0%) for both ileal and cecal contents for all groups assessed (Figure 5). Actinobacteria (0.4–2.2%) was enriched in ileal contents, and Tenericutes (0.8–3.1%) was enriched in cecal contents for all groups.

*Lactobacillus* was the predominant genus in the ileum for all groups except for the VX – Amprol contact group which had a higher abundance of *Streptococcus* compared to all treatment groups (Figure 5). The group with the highest abundance of *Lactobacillus* in the ileum was the PC group (54.2%). For the VX groups, the proportion of *Lactobacillus* was higher in the VX group (37.1% for directs and 26.6% for contacts) than in the VX + Amprol group (17.0% for directs and 9.5% for contacts) in ileal contents. Additionally, a higher abundance of *Clostridium* was observed in the VX group (15.4% for direct and 18.8% for contact) compared to the VX + Amprol group (10% for direct and 10% for contact). The VX + Amprol group was dominated by *Turicibacter* in the ileum compared to all other groups (24.6% for directs and contacts).

The dominant genera in the cecal contents at the genus level were *Faecalibacterium*, *X*. *Ruminococcus*, and *Lactobacillus* (Figure 5). The highest abundance of *Faecalibacterium* was in the VX + Amprol group (14.7% for directs and 15.8% for contacts) followed by the NC (10.9%) and VX contacts (11.0%) in cecal contents. However, X. *Ruminococcus* abundance was elevated for the VX contact group (11.5%) and VX + Amprol – contact group (10.2%) compared to the VX direct group (8.3%) and VX + Amprol – direct group (7.8%). *Clostridium* abundance in the cecal contents was highest in the VX – contact group (4.1%) compared to all treatment groups.

**Figure 6:**
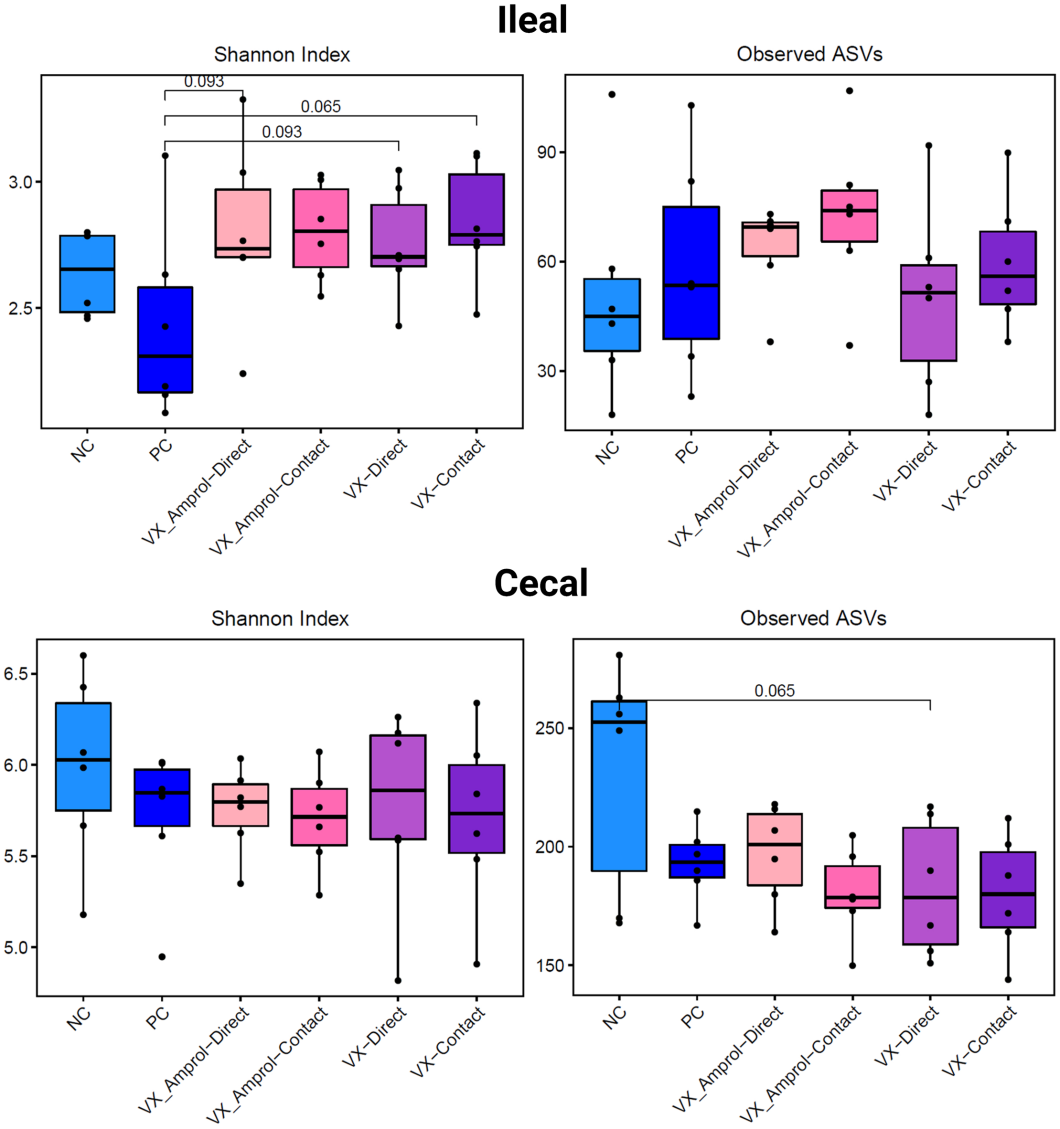
Alpha diversity of ileal and cecal contents collected at d29 (6d post-challenge). Alpha diversity was measured using Shannon Index (left) and number of Observed ASVs (right). Statistical comparison was made using the two-tailed Wilcoxon signed-rank test between two groups (*p* < 0.10) (Created with BioRender.com).

**Figure 7:**
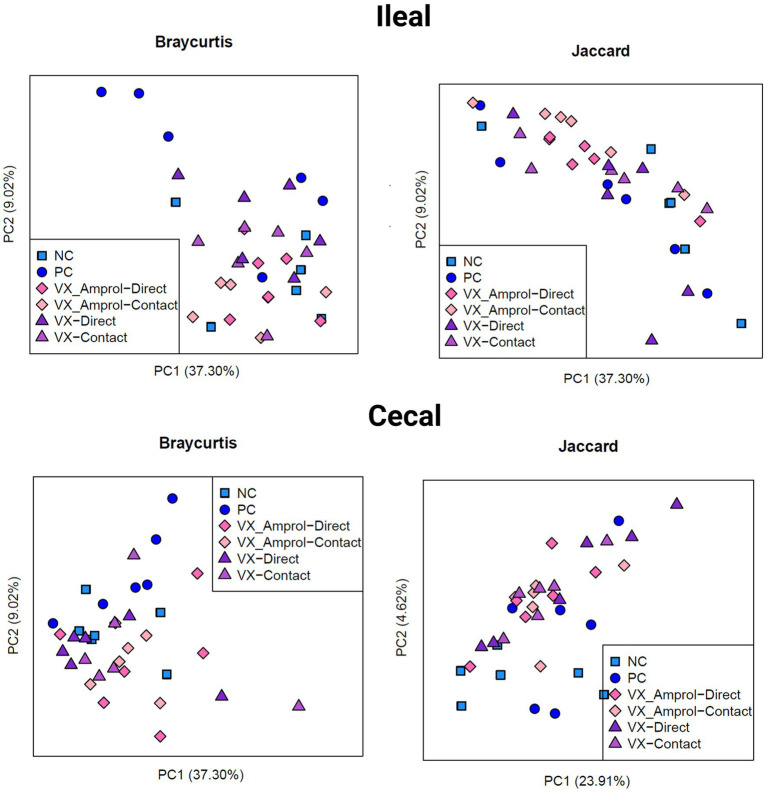
Beta diversity of ileal and cecal contents collected at d29 (6d post-challenge). Beta diversity was evaluated using Bray Curtis (left) and Jaccard (right) distances. The outputs of diversity were visualized using the “ggplot2” package in R (v4.1.2). Analysis was conducted using an analysis of similarity (ANOSIM) (Created with BioRender.com).

The results showed that ileal and cecal contents in the PC group did not exhibit a different bacterial diversity and structure, including alpha and beta diversity, when compared to NC (Figures 6, 7). Alpha diversity was measured using the Shannon Index and the number of observed ASVs. There were no significant *(p >* 0.10) differences for alpha diversity in ileal or cecal contents across vaccinated groups (Figure 6). However, the PC group had significantly (*p* < 0.10) lower alpha diversity (Shannon Index) in the ileal contents compared to all vaccinated groups, except for the VX + Amprol contact group. In the ceca, the NC group had significantly (*p* = 0.065) higher observed ASVs compared only to the VX – direct group. No distinct clusters were observed between the cecum and ileum-based Bray-Curtis and Jaccard distances across all groups (Figure 7).

**Figure 8:**
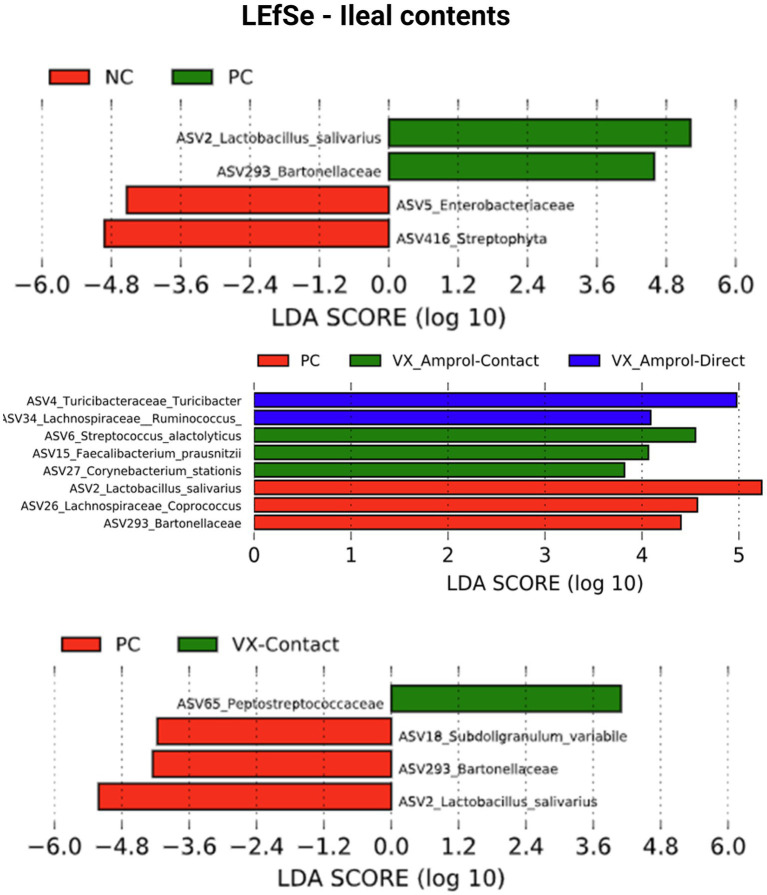
Linear discriminant analysis effect size (LEfSe) for negative control (NC) and positive control (PC); PC, VX + Amprol-Contact, and VX + Amprol-Direct; and PC and VX-Contact in ileal contents at the genus level. LEfSe was used to identify the signature bacteria associated with the ileal contents. LDA score>2 was used as a criterion for judging the significant effect size (Created with BioRender.com).

**Figure 9:**
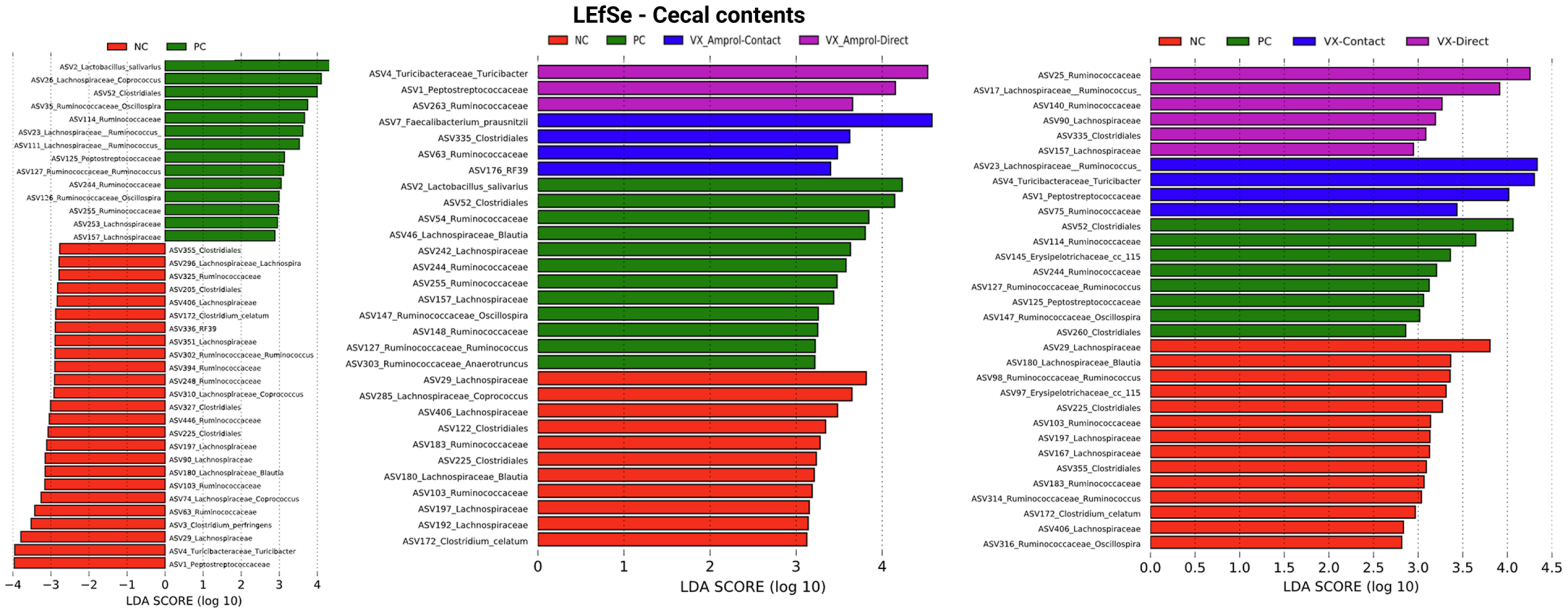
Linear discriminant analysis effect size (LEfSe) on effect post-challenge of *Eimeria meleagrimitis* vaccination with and without Amprol intervention in turkey cecal bacterial populations at the genus level for negative control (NC) and positive control (PC); PC, VX + Amprol-Contact, and VX + Amprol-Direct; and PC, VX-Contact and VX-Direct. LEfSe was used to identify the signature bacteria associated with the growth stages and intestinal segments. LDA score>2 was used as a criterion for judging the significant effect size (Created with BioRender.com).

Linear discriminant analysis Effect Size (LEfSe) was employed to identify bacterial biomarkers for each group. In the PC group, *Lactobacillus salivarius* (ASV2) was enriched in ileal and cecal content (Figures 8, 9). *Faecalibacterium prausnitzii* (ASV15 in ileal and ASV7 in cecal) was enriched in the cecal and ileal community of the VX + Amprol – contact group, while *Turicibacter* (ASV4) was overrepresented in VX + Amprol – direct group in ileal and cecal contents (Figures 8, 9). *Pepetostreptococcaceae* (ASV65) was only enriched in VX – contact group ileal contents (Figure 8), while *Ruminococcaceae* (ASV25) and *Lachnospiraceae_Ruminococcus* (ASV23) were greater in VX – direct and VX – contact group cecal contents, respectively (Figure 9).

## 4. Discussion

The importance of proper uptake of a live *E. meleagrimitis* vaccine candidate at hatch by turkey poults was demonstrated in the present study by comparing the difference in fecal OPG between contacts and directs of the VX group and between contacts of the VX and VX + Amprol group. Directs in the VX groups had attenuated shedding compared to the contact counterparts. Only numerical differences in BW or BWG between the vaccinated treatment groups were observed suggesting that this strain of *E. meleagrimitis* is relatively non-pathogenic, especially considering the “contact” poults were not directly vaccinated and the number of oocysts that were ingested was not controlled. Since susceptibility to *Eimeria* spp. infection increases with age, and the severity is associated with the number of oocysts ingested, a negative impact on performance was anticipated for contact poults, especially those that did not receive intermittent amprolium treatment.

It is important to note that there was apparent cross-contamination that occurred in the NC group which was reflected by litter and fecal OPG late in the study. Sporulated oocysts are incredibly resilient and although steps were taken to prevent cross contamination in the facility, dust and dander may have been a factor (29). Husbandry for both control groups was conducted before the vaccinated groups with showers being required between each check. Coccidiostat inclusion will be considered for the NC group in future studies.

Interestingly, the contact and directs in the VX group had improved gut integrity 6d post-challenge compared to all other treatments and were also numerically heavier than those that received amprolium in the drinking water from d0-14. Even though there were no differences in lesion scores between VX groups, the serum FITC-d levels of the VX + Amprol group were similar to the PC groups, which may suggest the timing of amprolium administration from d10-d14 was potentially too early. In the field, amprolium is generally administered in the diet ~d16 to mitigate performance losses associated with live coccidiosis vaccination at hatch. However, this practice is not accepted by all countries including many in Europe (30). Nevertheless, we hypothesize that arresting *E. meleagrimitis* development shortly after initiation of the second cycle by administering amprolium in the drinking water would have beneficial effects overall. A more comprehensive study is currently underway to validate these results.

This present study provides an initial evaluation of the effects of *E. meleagrimitis* and amprolium on the gut microbiome, specifically the ileal and cecal microbiome of turkey poults. *Eimeria* spp. infection impedes digestion and absorption of nutrients by impairing the intestinal barrier function, causing bacterial translocation, and disrupting gut homeostasis (31). The gut microbiome influences host performance and resistance to enteric pathogens (32), and the effects of the gut microbiome on the overall performance and health of chickens have been previously described (33–35). However, the microbiome of chickens and turkeys is only 16–19% similar at the genus level, indicating distinct variations between the two avian species (35). Several investigators have assessed the impact of coccidiosis on the gut microbiome of chickens (36, 37). In contrast, research evaluating the effect of live coccidiosis vaccination and anticoccidial drugs on the turkey gut microbiome is lacking.

In the current study, there was increased heterogeneity in the microbiome composition of cecal contents compared to the ileal contents. These results aligned with previous findings described by D’Andreano et al. (38), who assessed the gut microbiome of different gut sections of hemorrhagic enteritis-infected turkeys. Furthermore, the lack of significant differences in alpha and beta diversity across treatment groups is similar to a report published by Macdonald et al. (39), who demonstrated that live coccidiosis vaccination did not affect alpha diversity in the ceca of broiler chickens. In the present study, there was an apparent shift in the microbiome composition at both the phylum and genus levels. For example, the *E. meleagrimitis* challenge at d23 increased the abundance of *Lactobacillus salivarius* in the ileum of the PC group compared to the NC and the vaccinated groups, which was unexpected. Interestingly, Latorre et al. (12) observed the same phenomenon in necrotic enteritis-challenged chickens. Bacteria in the small intestine compete with the host to acquire and utilize amino acids, whereas the bacteria in the ceca capitalize on those amino acids or nutrients that bypass the small intestine (40). Perhaps the abundance of *Lactobacillus* is associated with the over-proliferation of lactobacilli due to the malabsorption of nutrients by the host associated with the *E. meleagrimitis* challenge. In contrast, amprolium administration was associated with a reduction in *Lactobacillus* in the ileum but an increase in *Turicibacter*, a known butyric acid producer associated with a normal/healthy gut in chickens (12). Although synthetic anticoccidials do not have antimicrobial effects, these drugs may indirectly affect the host’s gut microbiome since they affect parasite metabolism after intracellular invasion. The complexity of the host-microbiota-protozoa interaction and its effects on host immune development and performance requires further investigation.

Based on the results from the present study, vaccination with a non-attenuated strain of *E. meleagrimitis* obtained from wild turkey feces induced a mild infection providing protective immunity with and without amprolium intervention, which affected gut integrity and shifted the ileal and cecal luminal microbiome in turkey poults. The impact of a bioshuttle program on the intestinal microbiome requires further investigation.

## 5. Conclusion

Vaccination with *E. meleagrimitis* obtained from wild turkey feces induced a mild infection providing protective immunity based on lesion scores and performance. Results indicated that amprolium intervention could be used to attenuate *E. meleagrimitis* oocyst shedding but additional studies are required to determine the effect of a bioshuttle on gut permeability and the microbiome in turkey poults.

## Data availability statement

The datasets presented in this study can be found in online repositories. The names of the repository/repositories and accession number(s) can be found in the article/Supplementary material.

## Ethics statement

The animal study was reviewed and approved by Animal care and handling procedures complied with the University of Arkansas Institutional Animal Care and Use Committee (Animal Use Protocol #21026).

## Author contributions

CT-P, DG, GT-I, LB, and JB conceptualized the study. JL, JC, RS-C, AF, and MC handled the methodology. MC and JZ were in charge of the software. LB, JB, BH, and GT-I validated the study. CT-P and DG performed the formal analysis. CT-P, DG, and GT-I prepared and wrote the original draft. XH-V, JB, DG, and GT-I contributed to the writing, reviewing, and editing of the final manuscript. DG, BH, and GT-I oversaw the project administration and funding acquisition. All authors contributed to the article and approved the submitted version.

## Funding

This project was funded by USDA Animal Health Awards (FY2021 and FY2022), and by USDA-NIFA Sustainable Agriculture Systems, Grant No. 2019–69012-29905. Title of Project: Empowering U.S. Broiler Production for Transformation and Sustainability USDA-NIFA (Sustainable Agriculture Systems): No. 2019–69012-29905.

## Conflict of interest

The authors declare that the research was conducted in the absence of any commercial or financial relationships that could be construed as a potential conflict of interest.

## Publisher’s note

All claims expressed in this article are solely those of the authors and do not necessarily represent those of their affiliated organizations, or those of the publisher, the editors and the reviewers. Any product that may be evaluated in this article, or claim that may be made by its manufacturer, is not guaranteed or endorsed by the publisher.
